# Mutation landscape of germline and somatic *BRCA1/2* in patients with high-grade serous ovarian cancer

**DOI:** 10.1186/s12885-020-6693-y

**Published:** 2020-03-12

**Authors:** Kyung Jin Eoh, Hye Min Kim, Jung-Yun Lee, Sunghoon Kim, Sang Wun Kim, Young Tae Kim, Eun Ji Nam

**Affiliations:** 1 Department of Obstetrics and Gynecology, Yongin Severance Hospital, Yonsei University College of Medicine, Yongin, South Korea; 2 Institute of Women’s Life Medical Science, Women’s Cancer Center, Department of Obstetrics and Gynecology, Yonsei Cancer Center, Yonsei University College of Medicine, Seoul, South Korea; 3 Department of Pathology, Yonsei University College of Medicine, Yongin Severance Hospital, Yongin, South Korea

**Keywords:** *BRCA1/2* mutation, High-grade serous ovarian cancer, Next-generation sequencing, Poly (ADP-ribose) polymerase

## Abstract

**Background:**

Poly (ADP-ribose) polymerase inhibitors targeting *BRCA1/2* mutations are available for treating patients with high-grade serous ovarian cancer. These treatments may be more appropriately directed to patients who might respond if the tumor tissue is additionally tested by next-generation sequencing with a multi-gene panel and Sanger sequencing of a blood sample. In this study, we compared the results obtained using the next-generation sequencing multi-gene panel to a known germline *BRCA1/2* mutational state determined by conventional Sanger sequencing to evaluate the landscape of somatic mutations in high-grade serous ovarian cancer tumors.

**Methods:**

Cancer tissue from 98 patients with high-grade serous ovarian cancer who underwent Sanger sequencing for germline *BRCA1/2* analysis were consecutively analyzed for somatic mutations using a next-generation sequencing 170-gene panel.

**Results:**

Twenty-four patients (24.5%) showed overall *BRCA1/2* mutations. Seven patients (7.1%) contained only somatic *BRCA1/2* mutations with wild-type germline *BRCA1/2*, indicating acquired mutation of *BRCA1/2*. Three patients (3.1%) showed reversion of germline *BRCA1* mutations. Among the 14 patients (14.3%) with both germline and somatic mutations in *BRCA1/2*, two patients showed different variations of *BRCA1/2* mutations. The next-generation sequencing panel test for somatic mutation detected other pathogenic variations including *RAD51D* and *ARID1A*, which are possible targets of poly (ADP-ribose) polymerase inhibitors. Compared to conventional Sanger sequencing alone, next-generation sequencing-based tissue analysis increased the number of candidates for poly (ADP-ribose) polymerase inhibitor treatment from 17.3% (17/98) to 26.5% (26/98).

**Conclusions:**

Somatic mutation analysis by next-generation sequencing, in addition to germline *BRCA1/2* mutation analysis, should become the standard of care for managing women with high-grade serous ovarian cancer to widen the indication of poly (ADP-ribose) polymerase inhibitors.

## Background

*BRCA1/2* mutational loss of function is a primary driver of epithelial ovarian cancer and is the basis of therapeutics targeting a synthetic lethality mechanism of poly (ADP-ribose) polymerase (PARP) inhibition in combination with *BRCA1/2* mutation or possibly other homologous recombination genetic deficiencies [1, 2].

Most patients evaluated in previous PARP inhibitor-related randomized trials showed germline *BRCA1/2* mutations [3]. However, the results of these studies may also be applicable to patients with somatic *BRCA1/2* mutations [4, 5]. In 2014, the PARP inhibitor olaparib (Lynparza™, AstraZeneca, Cambridge, UK) was approved for treating patients with relapsed ovarian cancer with germline *BRCA1/2* mutations by the US Food and Drug Administration and European Medicines Agency and for patients with somatic *BRCA1/2* mutations by the European Medicines Agency [6].

In high-grade serous ovarian cancer (HGSOC), which comprises the majority of epithelial ovarian cancer cases, germline and somatic mutations in *BRCA1/2* are detected in 17–25% of patients, with somatic mutations representing 18–30% of all *BRCA1/2* mutations [7–9]. Analysis of ovarian cancer tissue from patients with HGSOC showed that loss of the normal copy of *BRCA1/2* occurs in most germline *BRCA1/2* mutations, indicating that this is an early event in HGSOC development [10].

In this study, we performed (i) next-generation sequencing (NGS) to determine the mutational state of *BRCA1/2* in ovarian cancer tissues from 98 consecutive patients with HGSOC; (ii) compared the results to the known germline *BRCA1/2* mutational state by conventional Sanger sequencing of blood samples; and (iii) determined the genetic landscape of somatic mutations in HGSOC tumors.

## Methods

### Study population

An electronic medical record review of patients treated for HGSOC at the Department of Obstetrics and Gynecology at the Severance Hospital of Yonsei University between January 2017 and February 2019 was carried out. Ninety-eight patients with HGSOC who were tested for both germline and somatic *BRCA1/2* mutations were included in the analysis. The medical record and pedigree of each patient were reviewed, and data including age at HGSOC diagnosis, family history of *BRCA1/2*-related cancer, history of primary breast cancer, residual disease after cytoreductive surgery, and survival status were collected. A patient was considered to have a family history of *BRCA1/2*-related cancer if there were one or more instances of ovarian, peritoneal, fallopian tube, breast, pancreas, or prostate cancer among first- or second-degree relatives. This study was reviewed and approved by our organization’s institutional review board and was performed in accordance with the ethical standards described in the Declaration of Helsinki.

### Genetic testing for germline BRCA1/2 mutations

All patients underwent in-house testing as previously reported [9]. Briefly, we identified all small base pair variations by Sanger sequencing on a 3730 DNA Analyzer with the BigDye Terminator v3.1 Cycle Sequencing Kit (Applied Biosystems, Foster City, CA, USA). Sequencing data were aligned against appropriate reference sequences and analyzed using Sequencher 5.3 software (Gene Codes Corp., Ann Arbor, MI, USA).

### Genetic testing for somatic mutations using NGS multi-gene panel

All the tissue used for the NGS analysis was obtained at the first time of taking cancer tissue, i.e. at the time of primary debulking surgery or diagnostic laparoscopy. Formalin-fixed paraffin-embedded (FFPE) sections (5-μm-thick) were deparaffinized in xylene, hydrated through graded alcohols to water, and stained with Gill’s hematoxylin. The slides were manually microdissected under a dissecting microscope using a scalpel point dipped in ethanol. The scraped material was washed in phosphate-buffered saline and digested in proteinase K overnight at 37 °C in ATL Buffer (Qiagen, Hilden, Germany). DNA and RNA were then isolated using the QIAamp DSP DNA FFPE extraction kit (cat # 60404) and RNeasy FFPE kit (cat # 73504) according to the manufacturer instructions.

Mutational and copy number analysis was carried out using the Illumina TST-170 panel, according to the manufacturer instructions (San Diego, CA, USA). The gene panels cover 170 cancer-related genes for mutational analysis and 59 genes for copy number analysis (Supplementary Table 1). For mutational analysis, FASTQ files were uploaded on Illumina’s BaseSpace software for variant interpretation. Only variants in coding regions and promoter regions or splice variants were retained. In addition, only variants present in < 1% of the population according to ExAC and 1000 Genomes databases, and which were present in > 5% of reads with a minimum read depth of 250, were retained. All retained variants were reviewed using reference websites [Catalogue of Somatic Mutations in Cancer (http://evs.gs.washington.edu/EVS/), Precision Oncology Knowledge Base (http://oncokb.org), and dbSNP (https://www.ncbi.nlm.nih.gov/snp)], and only pathogenic variants were selected. In copy number analysis, genes showing greater than 2-fold change compared to the average level were considered to have undergone amplification. Genes showing a lower than 0.7-fold change compared to the average levels were considered to exhibit significant copy number loss. Fusion and splice variants were detected by RNA analysis workflow in the TST-170 panel. RNA was converted to cDNA in the first step, and the remaining steps of NGS library preparation, hybrid-capture based enrichment, and sequencing were similar to the workflow of the DNA analysis module of TST-170 except for the hybrid-capture probes for 55 genes included in the RNA analysis workflow. Data analysis for fusion and splice variants was performed with TST-170 Local App provided by Illumina. Specifically, Manta was used for the fusion variant calling. For splice variant calling, Illumina’s RNA Splice Variant Calling software was used.

### Statistical analyses

IBM SPSS version 23 for Windows (SPSS Inc., Chicago, IL, USA) was used for statistical analysis. The Kolmogorov–Smirnov test was used to validate standard normal distribution assumptions. Pearson’s chi-square test, Fisher’s exact test, Student’s *t*-test and Mann–Whitney *U*-test were used for univariate analysis. Survival outcomes were determined using Kaplan–Meier survival analysis.

## Results

### Study population

**Fig. 1:**
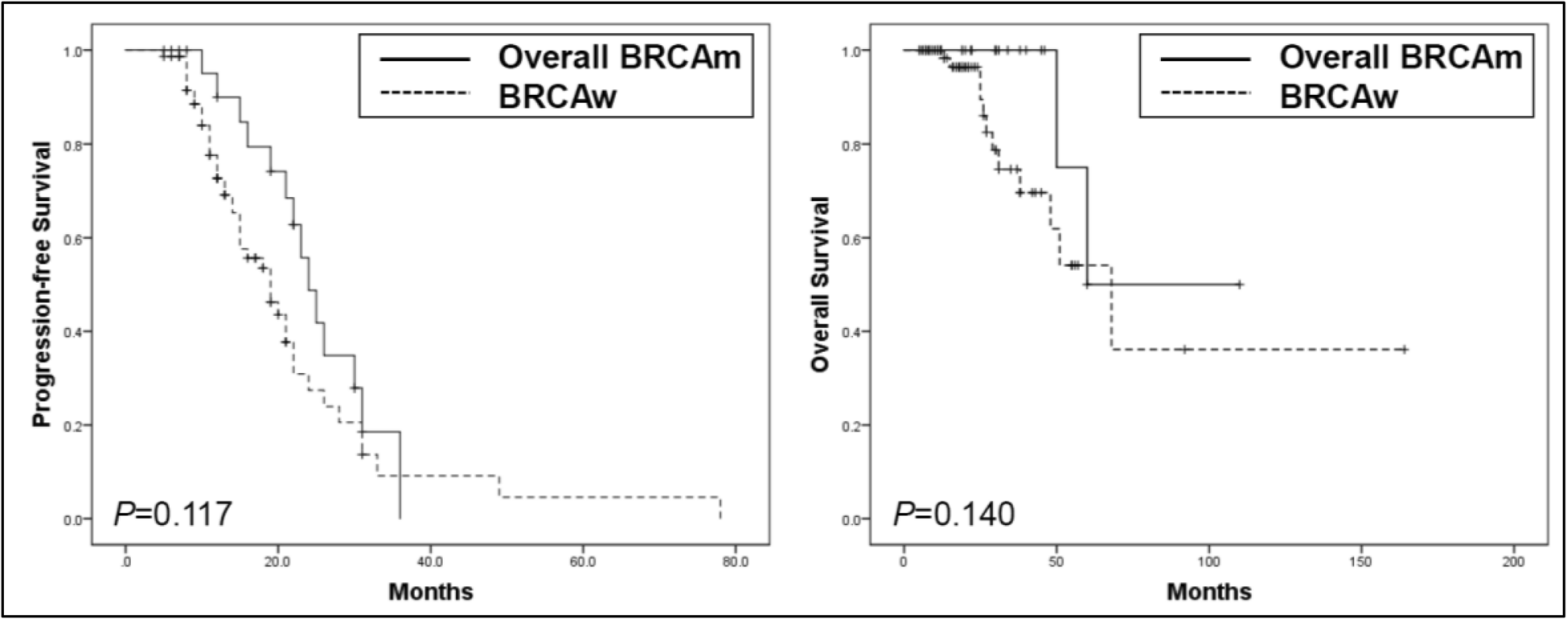
Comparison of survival between overall *BRCA* mutation and *BRCA* wild-type in the Kaplan-Meier curve. BRCAm, *BRCA* mutation; BRCAw, *BRCA* wild-type

Patient characteristics are shown in Table 1. Of the 98 patients, 46 patients received neoadjuvant chemotherapy (NAC) after diagnostic laparoscopy. There was no difference in the proportion of patients who treated with NAC between the overall *BRCA1/2* mutation group and the *BRCA1/2* wild-type group. All the patients receive platinum-based chemotherapy, and PARP inhibitor was not used. Patients with overall *BRCA1/2* mutations tended to show a higher rate of *BRCA1/2*-related family history and breast cancer history compared to patients with wild-type *BRCA1/2*. However, no factors showed a significant difference. Overall *BRCA1/2* mutation appeared to be correlated with a better prognosis than wild-type *BRCA1/2*, which was comparable to the results of our previous study; however, this tendency was not significant, likely because of the small number of patients (Fig. 1) [11].

**Table 1:** Patient characteristics

	Overall BRCAm (*n* = 24)		BRCAw (*n* = 74)	*P*
	Germline BRCAm (*n* = 17)	Only Somatic BRCAm (n = 7)		
Age	53 (42–74)	54 (48–77)	56 (22–78)	0.353
Stage				
I	0	0	1 (1.4)	0.838
II	1 (5.9)	0	3 (4.1)	
III	7 (41.2)	3 (42.9)	35 (47.3)	
IV	9 (52.9)	4 (57.1)	35 (47.3)	
Breast cancer	2 (11.8)	0	1 (1.4)	0.084
BRCA-related FHx	4 (23.5)	3 (42.9)	9 (12.2)	0.05
NGR	10 (58.8)	6 (85.7)	42 (56.8)	0.39
NAC	10 (58.8)	4 (57.1)	32 (43.2)	0.242

*BRCAm* BRCA mutation, *BRCAw* BRCA wild type, *FHx* family history, *NGR* no gross residual disease, *NAC* neoadjuvant chemotherapy

### Frequency and spectrum of germline and somatic BRCA1/2 mutation

**Fig. 2:**
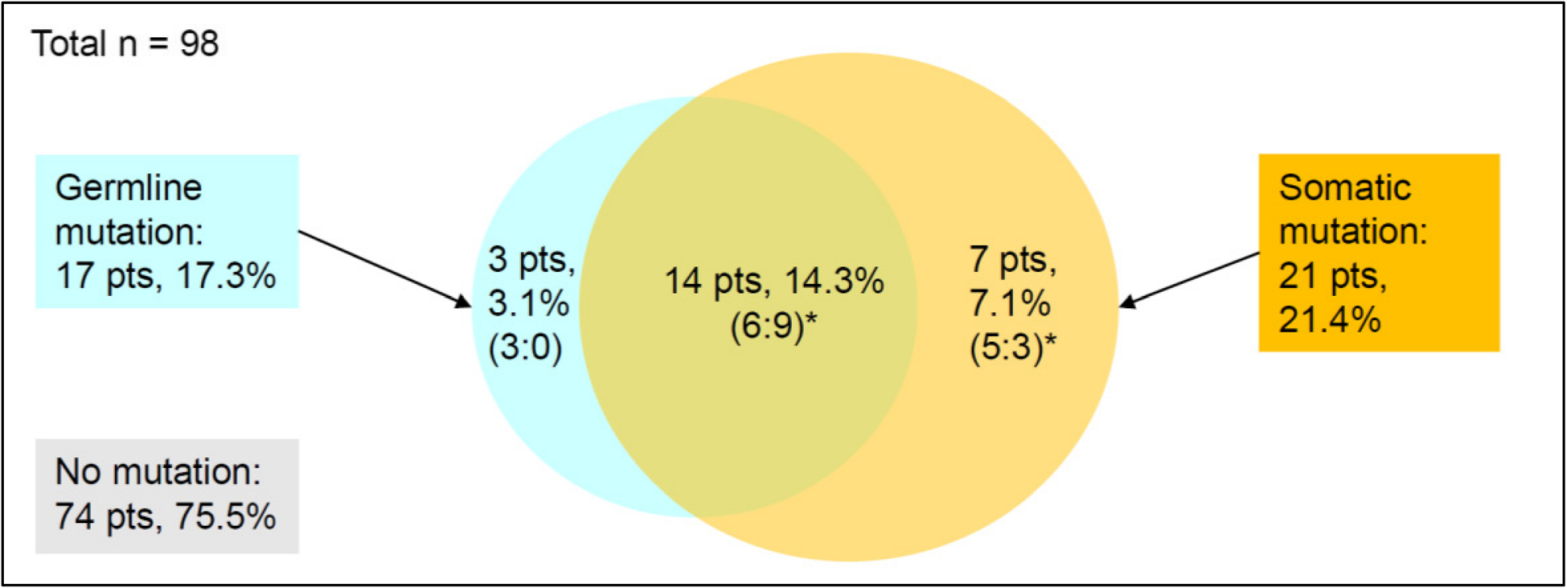
Distribution of germline and somatic *BRCA1/2* mutations. Pts, patients

Figure 2 shows the distribution of germline and somatic *BRCA1/2* mutations in this population. Twenty-four (24.5%) of the 98 patients had either germline or somatic *BRCA1/2* mutations. Among the 24 patients with mutations in BRCA1 or BRCA2, 14 showed both germline and somatic mutations. However, three and seven patients contained only germline and only somatic mutations, indicating reversion (Reversion #1–3) and acquired mutation (Acquired #1–#7) of *BRCA1/2*, respectively (Table 2). Interestingly, even among the 14 patients who had both germline and somatic mutations, two showed variations in *BRCA1/2* (Replace #1–#2). The inconsistent *BRCA1/2* status is presented in Table 2.

**Table 2:** Inconsistent germline and somatic BRCA1/2 variations

	Germline BRCA mutation				Somatic BRCA mutation				
		AA variation	Sequence variation	Mutation type		AA variation	Sequence variation	Mutation type	VAF
Replace #1	*BRCA2*	p.S93Ifs*8	c.276dupA	Frameshift	*BRCA1*	p.K654Sfs*47	c.1961delA	Frameshift	2.11
					*BRCA2*	p.I605Yfs*9	c.1806delA	Frameshift	24.65
Replace #2	*BRCA1*	p.W1815*	c.5445G > A	Nonsense	*BRCA1*	p.W1836*	c.5508G > A	Nonsense	54.33
Reversion #1	*BRCA1*	p.S308*fs	c.922_924delAGCinsT	Frameshift	–				
Reversion #2	*BRCA1*		c.5467 + 1G > A	Intervening sequence	–				
Reversion #3	*BRCA1*		c.212 + 1G > T	Intervening sequence	–				
Acquired #1	–				*BRCA2*	p.E1299V	c.3896A > T	Missense	5.54
Acquired #2	–				*BRCA1*	p.5308Tfs*6	c.923delG	Frameshift	54.73
Acquired #3	–				*BRCA1*	p.F1177Cfs*7	c.3530_3540del	Frameshift	27.84
Acquired #4	–				*BRCA1*	p.K1183R	c.3548A > G	Missense	23.74
Acquired #5	–				*BRCA1*	p.K654Sfs’47	c.1961delA	Frameshift	2.11
					*BRCA2*	p.I605Yfs’9	c.1806delA	Frameshift	24.77
Acquired #6	–				*BRCA2*	p.I605Nfs’11	c.1805_1805insA	Frameshift	2.34
Acquired #7	–				*BRCA1*	p.T499Kfs*4	c.1496delC	Frameshift	40.87

*AA* amino acid, *VAF* variant allele frequency, *VOUS* variants of uncertain significance

### Landscape of somatic mutations shown in NGS multi-gene panel

**Fig. 3:**
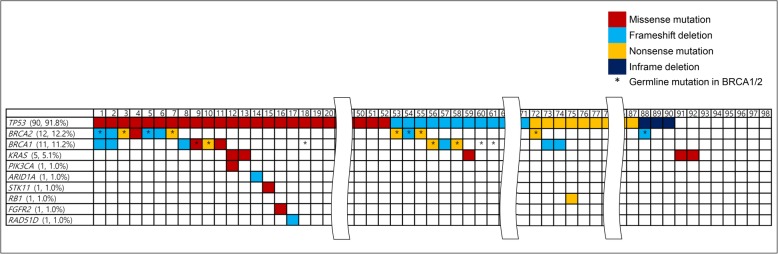
Landscape of somatic mutations and germline BRCA1/2 mutations in 98 patients with HGSOC. HGSOC, high-grade serous ovarian cancer

The landscape of somatic mutations of the included patients is shown in Fig. 3. Ten mutated genes were detected in 92 (93.9%) of the 98 patients: *TP53*, *BRCA2*, *BRCA1*, *KRAS*, *ARID1A*, *RB1*, *PIK3CA*, *STK11*, *FGFR2*, and *RAD51D*. Among them, four genes, *TP53*, *BRCA1*, *BRCA2*, and *KRAS*, were detected in multiple patients. *TP53* mutation was observed in 90 (91.8%) patients, including missense mutation (52, 57.8%), frameshift (19, 21.1%), nonsense mutation (16, 17.8%), and in-frame deletion (3, 3.3%). *BRCA1* and *BRCA2* mutations were detected in 11 (11.2%) and 12 (12.2%) patients, respectively. All patients who had *BRCA1* or *BRCA2* mutations showed TP53 mutation. KRAS mutation was detected in 5 patients (5.1%). Additionally, *BRCA1*/2, *ARID1A*, and *RAD51D* mutations, which are introduced by homologous recombination, may be targets of PARP inhibitors [12, 13]. The median depth of NGS sequencing was 880.5 (range, 280–1337). All information on patients enrolled in this study is presented in Supplementary Table 2.

Somatic mutations were compared to those in 316 patients with serous ovarian cancer from The Cancer Genome Atlas (Supplement Figure 1). *TP53* mutation was detected in 88% of patients (277 patients), which was comparable to the value in our data. However, *BRCA1* and *BRCA2* were observed in 4% (12 patients) and 3% of patients (11 patients); these values were considerably smaller than those in our data.

## Discussion

### Principal findings

In this study, we compared the germline and somatic *BRCA1/2* mutation status of 98 patients with HGSOC. Twenty-four (24.5%) of the 98 patients had either germline or somatic *BRCA1/2* mutations. Three of 17 patients (17.6%) showed restored *BRCA1/2* mutation, and seven of 81 patients (8.6%) exhibited acquired *BRCA1/2* mutations. These data indicate that among the patients who were negative for germline *BRCA1/2* mutation, approximately 10% may have only somatic *BRCA* mutations without germline mutation.

## Results

NGS-based multi-gene panel testing of ovarian cancer tissue allows for the identification of somatic mutations that are not detected by blood-based examination and are often not identified at low allele frequencies by Sanger sequencing of tumor samples. The tumor assay used in this study showed that 93.9% (92/98) of patients with somatically mutated tumors, including *TP53*, *BRCA1*, *BRCA2*, *KRAS*, *ARID1A*, *RB1*, *PIK3CA*, *STK11*, *FGR2*, and *RAD51D*, were not adequately detected by Sanger sequencing and *BRCA1/2* testing of clinical FFPE sections. Furthermore, the ability to determine the mutational status of 170 cancer genes simultaneously provides insight into the co-occurrence patterns of mutations, additional oncogenic drivers, and intra- or inter-tumor heterogeneity, and is useful for identifying homologous recombination and DNA repair genes beyond *BRCA1/2* which may be involved in the response to PARP inhibitors. *ARID1A* and *RAD51D* mutations were found in 2 patients, demonstrating that these patients are possible candidates for PARP inhibitor treatment [12, 13]. Thus, compared to conventional Sanger sequencing alone, NGS-based tissue analysis increased the number of candidates for PARP inhibitor treatment from 17.3% (17/98) to 26.5% (26/98).

*TP53* mutation was found in 90 (91.8%) patients. The overall frequency of *TP53* mutation in the 316 patients from The Cancer Genome Atlas 2011 study was 88.0% (277/316), which is comparable to our results (Supplement Figure 1). All patients with *BRCA1/2* mutation showed *TP53* mutation, indicating that *BRCA1/2* mutation is an earlier event than *TP53* mutation. The correlation between *BRCA1/2* and TP53 observed in this study is consistent with the results of previous studies evaluating patients with breast cancer [14].

### Clinical implications

In the previous study, patients with ovarian cancer who did not carry germline *BRCA1/2* mutations also responded to PARP inhibitors, suggesting that the broader dysfunction of genes, such as a homologous recombination-deficient phenotype, is important [15]. As initially reported in a previous phase II study of olaparib, objective responses were confirmed in 41% (7/17) of patients with ovarian cancer with germline *BRCA* mutations and 24% (11/46) of patients without germline mutations [16]. Remarkably, in responders belonging to the latter group, *BRCA1/2* somatic mutations were detected. Subsequent studies of olaparib (Study 19) and rucaparib (ARIEL 2 and Study 10) confirmed that *BRCA*-mutated patients derived the most significant clinical benefit from PARP inhibitor treatment and showed no differences in responsiveness to PARP inhibitors between germline and somatic *BRCA*-mutated HGSOC [17–19]. Both *BRCA1*, located on chromosome 17 (17q21), and *BRCA2*, on chromosome 13 (13q12.3), are very large, and exon 11 of both is thought to encode relevant protein domains as mutations in these regions are highly pathogenic [10, 20]. However, because of the gene length and domain complexity, pathogenic mutations may occur anywhere and can be highly variable as well as depend on ethnicity [21, 22].

Since the introduction of PARP inhibitors as the first targeted therapy, ovarian cancer diagnosis has involved not only standard morphological and immunophenotypic evaluation of cancer samples, but also detailed genotyping and mutational profiling. Additionally, an understanding of the mutational status in genes essential for drug sensitivity and resistance is necessary to ensure effective treatment of ovarian cancer. As more studies are conducted and targeted therapeutics become available, genomic analysis for cancer diagnosis and treatment will benefit a large number of patients who currently have unmet medical needs.

### Research implications

Further studies are required to determine whether the extent and duration of benefit in patients with germline and somatic *BRCA1/2* mutations are equivalent. Additionally, the appropriate time for obtaining the tumor tissue for somatic mutational analysis must be determined. Specifically, whether previously archived FFPE sections miss patients whose somatic mutations are acquired later in their pathway of cancer should be evaluated. Notably, 100% of germline and 83% of somatic loss-of-function mutations showed biallelic inactivation and were predominantly clonal, suggesting that loss of function of *BRCA* can occur early in the development of HGSOC [23]. This finding indicates that retesting for somatic *BRCA1/2* mutations using fresh biopsies at each relapse may not be informative, although data from ARIEL 2 showed controversial results regarding this point [19].

Low-grade serous ovarian cancer (LGSOC) was known to have a high prevalence of KRAS and BRAF mutations, but a low prevalence of TP53 mutations [24]. In our study, five cases showed KRAS mutation. However, three out of the five patients had simultaneous TP53 mutations as well. Additionally, contrary to the previous report, all the KRAS-mutated patients were diagnosed with HGSOC, not LGSOC. We hope to be able to report a further study that investigates the impact of the results of NGS panel on the conventional histologic diagnosis reversely, showing how much proportion of the conventional histologic diagnosis would be changed reflecting the results of NGS.

### Strengths and limitations

This is the first study to compare the germline and somatic *BRCA1/2* mutation status in patients of Asian ethnicity, which may guide future research of Asian patients with HGSOC. The current study had some limitations; it included a small number of patients and was performed in a single center. Additionally, NGS-based tests were not conducted as prospective schedules. Therefore, the timing of tests was variable among patients and the exact timing of the acquired or reversion of *BRCA1/2* mutations could not be investigated. Also, technical challenges in identifying the mutations in tumors, such as difficulty in detecting mutation from archival tumor specimen and issues related with intratumoral heterogeneity, might be criticized as limitations of our study. Additionally, we could not show whether the “replace” or “reversion” cases really have the function of replaced or reversed BRCA1/2 protein, respectively.

## Conclusions

The effectiveness of PARP inhibitors likely extends beyond the treatment of germline *BRCA1/2* mutations to include homologous recombination deficiency in patients with HGSOC. NGS-based somatic mutation analysis, as well as germline *BRCA1/2* mutation analysis, should become the standard of care for managing women with ovarian cancer to widen the indication of PARP inhibitors.

## Supplementary information

**Additional file 1: Supplement Table 1.** Table of 170 genes evaluated in the NGS multi-gene panel in this study. The gene panels cover 170 cancer-related genes for mutational analysis and 59 genes for copy number analysis.

**Additional file 2: Supplement Table 2.** All information on patients enrolled in this study.

**Additional file 3: Supplement Figure 1.** Landscape of somatic mutations detected in this study in the TCGA database (316 patients with serous ovarian cancer). Somatic mutations were compared to those in 316 patients with serous ovarian cancer from The Cancer Genome Atlas.

## Data Availability

The datasets used and/or analysed during the current study available from the corresponding author on reasonable request.

## Abbreviations

FFPE: Formalin-fixed paraffin-embedded
HGSOC: High-grade serous ovarian cancer
NAC: Neoadjuvant chemotherapy
NGS: Next-generation sequencing
PARP: Poly (ADP-ribose) polymerase
