# Supplementary material for: Mutation landscape of germline and somatic BRCA1/2 in patients with high-grade serous ovarian cancer
Source: BMC Cancer. 2020 Mar 12;20:204. doi: 10.1186/s12885-020-6693-y (PMC7069205; doi:10.1186/s12885-020-6693-y)
Supplement: Supplementary file 1 — Additional file 1: Supplement Table 1. Table of 170 genes evaluated in the NGS multi-gene panel in this study. The gene panels cover 170 cancer-related genes for mutational analysis and 59 genes for copy number analysis. [file 12885_2020_6693_MOESM1_ESM.docx]

Supplement Table 1. Table of 170 genes evaluated in the NGS multi-gene panel in this study.

| **Small nucleotide variant and indels (from DNA)** | | | | | | | | | | |
| --- | --- | --- | --- | --- | --- | --- | --- | --- | --- | --- |
| *AKT1* | *BRIP1* | *CREBBP* | *FANCI* | *FGFR2* | *JAK3* | *MSH3* | *PALB2* | *RAD51B* | | *TET2* |
| *AKT2* | *BTK* | *CSF1R* | *FANCL* | *FGFR3* | *KDR* | *MSH6* | *PAX3* | *RAD51C* | | *TMPRSS2* |
| *AKT3* | *CARD11* | *CTNNB1* | *FBXW7* | *FGFR4* | *KIT* | *MTOR* | *PAX7* | *RAD51D* | | *TP53* |
| *ALK* | *CCND1* | *DDR2* | *FGF1* | *FLT1* | *KMT2A* | *MUTYH* | *PDGFRA* | *RAD54L* | | *TSC1* |
| *APC* | *CCND2* | *DNMT3A* | *FGF2* | *FLT3* | *KRAS* | *MYC* | *PDGFRB* | *RB1* | | *TSC2* |
| *AR* | *CCNE1* | *EGFR* | *FGF3* | *FOXL2* | *MAP2K1* | *MYCL1* | *PIK3CA* | *RET* | | *VHL* |
| *ARID1A* | *CD79A* | *EP300* | *FGF4* | *GEN1* | *MAP2K2* | *MYCN* | *PIK3CB* | *RICTOR* | | *XRCC2* |
| *ATM* | *CD79B* | *ERBB2* | *FGF5* | *GNA11* | *MCL1* | *MYD88* | *PIK3CD* | *ROS1* | |  |
| *ATR* | *CDH1* | *ERBB3* | *FGF6* | *GNAQ* | *MDM2* | *NBN* | *PIK3CG* | *RPS6KB1* | |  |
| *BAP1* | *CDK12* | *ERBB4* | *FGF7* | *GNAS* | *MDM4* | *NF1* | *PIK3R1* | *SLX4* | |  |
| *BARD1* | *CDK4* | *ERCC1* | *FGF8* | *HNF1A* | *MET* | *NOTCH1* | *PMS2* | *SMAD4* | |  |
| *BCL2* | *CDK6* | *ERCC2* | *FGF9* | *HRAS* | *MLH1* | *NOTCH2* | *PPP2R2A* | *SMARCB1* | |  |
| *BCL6* | *CDKN2A* | *ERG* | *FGF10* | *IDH1* | *MLLT3* | *NOTCH3* | *PTCH1* | *SMO* | |  |
| *BRAF* | *CEBPA* | *ESR1* | *FGF14* | *IDH2* | *MPL* | *NPM1* | *PTEN* | *SRC* | |  |
| *BRCA1* | *CHEK1* | *EZH2* | *FGF23* | *INPP4B* | *MRE11A* | *NRAS* | *PTPN11* | *STK11* | |  |
| *BRCA2* | *CHEK2* | *FAM175A* | *FGFR1* | *JAK2* | *MSH2* | *NRG1* | *RAD51* | *TERT* | |  |
| **Copy number variation (from DNA)** | | | | | | | | | | |
| *AKT2* | *BRCA2* | *CHEK1* | *ERCC2* | *FGF5* | *FGF14* | *FGFR4* | *MDM4* | *NRG1* | *RAF1* | |
| *ALK* | *CCND1* | *CHEK2* | *ESR1* | *FGF6* | *FGF19* | *JAK2* | *MET* | *PDGFRA* | *RET* | |
| *AR* | *CCND3* | *EGFR* | *FGF1* | *FGF7* | *FGF23* | *KIT* | *MYC* | *PDGFRB* | *RICTOR* | |
| *ATM* | *CCN21* | *ERBB2* | *FGF2* | *FGF8* | *FGFR1* | *KRAS* | *MYCL1* | *PIK3CA* | *RPS6KB1* | |
| *BRAF* | *CDK4* | *ERBB3* | *FGF3* | *FGF9* | *FGFR2* | *LAMP1* | *MYCN* | *PIK3CB* | *TFRC* | |
| *BRCA1* | *CDK6* | *ERCC1* | *FGF4* | *FGF10* | *FGFR3* | *MDM2* | *NRAS* | *PTEN* |  | |
| **Fusions and splice variants (from RNA)** | | | | | | | | | | |
| *ABL1* | *BRAF* | *EML4* | *ETV4* | *FGFR4* | *KIF5B* | *MYC* | *NTRK3* | *PIK3CA* | *TMPRSS2* | |
| *AKT3* | *BRCA1* | *ERBB2* | *ETV5* | *FLI1* | *KIT* | *NOTCH1* | *NTRK3* | *PPARG* |  | |
| *ALK* | *FRCA2* | *ERG* | *EWSR1* | *FLT1* | *KMT2A* | *NOTCH2* | *PAX3* | *RAF1* |  | |
| *AR* | *CDK4* | *ESR1* | *FGFR1* | *FLT3* | *MET* | *NOTCH3* | *PAX7* | *RET* |  | |
| *AXL* | *CSF1R* | *ETS1* | *FGFR2* | *JAK2* | *MLLT3* | *NRG1* | *PDGFRA* | *ROS1* |  | |
| *BCL2* | *EGFR* | *ETV1* | *FGFR3* | *KDR* | *MSH2* | *NTRK1* | *PDGFRB* | *RPS6KB1* |  | |
