# Supplementary material for: Mutation landscape of germline and somatic BRCA1/2 in patients with high-grade serous ovarian cancer
Source: BMC Cancer. 2020 Mar 12;20:204. doi: 10.1186/s12885-020-6693-y (PMC7069205; doi:10.1186/s12885-020-6693-y)
Supplement: Supplementary file 3 — Additional file 3: Supplement Figure 1. Landscape of somatic mutations detected in this study in the TCGA database (316 patients with serous ovarian cancer). Somatic mutations were compared to those in 316 patients with serous ovarian cancer from The Cancer Genome Atlas. [file 12885_2020_6693_MOESM3_ESM.docx]

Supplement Figure 1. Landscape of somatic mutations detected in this study in the TCGA database (316 patients with serous ovarian cancer).

Downloaded from: http://www.cbioportal.org/results/oncoprint?Action=Submit&RPPA_SCORE_THRESHOLD=2.0&Z_SCORE_THRESHOLD=2.0&cancer_study_list=ov_tcga&case_set_id=ov_tcga_sequenced&data_priority=0&gene_list=TP53%2520BRCA2%2520BRCA1%2520KRAS%2520PIK3CA%2520ARID1A%2520STK11%2520RB1%2520FGFR2%2520RAD51D&geneset_list=%20&genetic_profile_ids_PROFILE_MUTATION_EXTENDED=ov_tcga_mutations&show_samples=false&tab_index=tab_visualize
